# 

**Published:** 1805-05-01

**Authors:** 


### To CORRESPONDENTS.

Communications are received from Dr. Kinglake, Mr. Forbes, Mr. Howard, Mr. Pearson, Mr. Waiblinger, Mr. Wilkinson, Mr. Dunning, Mr. Highmore, Sir John Sinclair, Bart. Dr. Walker, Mr. Simpson, Mr. Jenkinson, D. L. and Mr. Bethune.

